# Dietary Sanguinarine Enhances Antioxidant Capacity and Intestinal Barrier Function in Yellow-Feathered Broilers via Modulation of Microbiota-Associated Butyrate Signaling

**DOI:** 10.3390/antiox15101317

**Published:** 2026-10-13

**Authors:** Xin Li, Chuanbin Chen, Lihua Li, Minfeng Ding, Wenyan Zhang, Weifen Li, Lanjiao Xu, Qi Wang

**Affiliations:** 1 Jiangxi Province Key Laboratory of Animal Nutrition and Feed, College of Animal Science and Technology, Jiangxi Agricultural University, Nanchang 330045, China; 2 Key Laboratory of Animal Feed and Nutrition of Zhejiang Province, Institute of Animal Nutrition and Feed Sciences, College of Animal Sciences, Zhejiang University, Hangzhou 310058, China

**Keywords:** phytogenic feed additive, short-chain fatty acids, GPR109A, Wnt/β-catenin signaling, oxidative stress, intestinal organoid

## Abstract

Sanguinarine (SA), a bioactive benzophenanthridine alkaloid, has emerged as a potential alternative to antibiotic growth promoters; however, its effects on intestinal redox homeostasis and epithelial regeneration remain incompletely understood. This study investigated whether dietary SA could improve growth performance and intestinal health in yellow-feathered broilers and explored the potential involvement of gut microbiota-associated metabolite signaling pathways. Broilers were fed diets supplemented with different concentrations of SA (0.5 or 0.7 mg/kg feed), with an antibiotic-supplemented group serving as a comparator. High-dose SA (0.7 mg/kg feed) improved body weight and average daily gain while reducing the feed-to-gain ratio. SA also alleviated intestinal barrier dysfunction, as evidenced by reduced circulating diamine oxidase, D-lactate, and lipopolysaccharide levels, together with increased jejunal expression of tight-junction proteins and *MUC2*. In parallel, SA enhanced systemic and intestinal antioxidant capacity, as indicated by increased total antioxidant capacity and superoxide dismutase activity and decreased malondialdehyde accumulation. These changes were accompanied by increased immunoglobulin levels, reduced expression of pro-inflammatory mediators, and enhanced expression of anti-inflammatory cytokines. Cecal microbiota analysis demonstrated that SA markedly reshaped the microbial community, with enrichment of *Faecalibacterium* and depletion of several potentially unfavorable taxa. These microbial alterations, which were associated with improved antioxidant and immune indices, were accompanied by a selective increase in cecal butyrate levels. Consistently, SA enhanced intestinal GPR109A expression and upregulated genes associated with Wnt/β-catenin signaling. In LPS-challenged intestinal organoids, SA restored organoid growth and budding capacity, reduced apoptosis, increased Ki67-positive cell proliferation, and restored the expression of genes associated with Wnt/β-catenin signaling. Collectively, these findings indicate that dietary SA improves growth performance and intestinal homeostasis in broilers by attenuating oxidative stress and inflammation, strengthening epithelial barrier function, and supporting epithelial regeneration. The microbiota-associated butyrate/GPR109A/Wnt/β-catenin signaling pathway may contribute to these protective intestinal effects.

## 1. Introduction

The restriction of growth-promoting antibiotics represents a major transition in modern animal production [1,2]. Although antibiotics have traditionally been used to improve feed efficiency and reduce the incidence of enteric diseases, their widespread use has raised persistent concerns regarding antimicrobial resistance, drug residues, and food safety [3,4]. With the gradual reduction or prohibition of growth-promoting antibiotics in animal production, there is an increasing demand for safe and effective feed additives [5,6]. In broiler production, the intestine not only plays a central role in nutrient digestion and absorption but also serves as a major interface among dietary components, the gut microbiota, and the host immune system [7,8]. Disruption of the intestinal barrier, coupled with excessive inflammation, oxidative stress, and microbial dysbiosis, can impair nutrient utilization and growth performance and increase susceptibility to disease [9]. Therefore, identifying antibiotic alternatives that improve intestinal homeostasis has become a key strategy for sustainable poultry production.

Yellow-feathered broilers constitute an important segment of poultry production in China because of their desirable meat quality and strong consumer demand [10,11]. Phytogenic feed additives, including natural plant and herbal extracts, have attracted increasing attention as potential alternatives to conventional antibiotic growth promoters because of their diverse biological activities [12,13]. Considerable attention has been directed toward Sanguinarine (SA), a benzophenanthridine alkaloid naturally sourced from *Macleaya cordata*, regarding its potential as an eco-friendly feed supplement. Its value is underscored by its potent antimicrobial, antioxidative, and anti-inflammatory properties, alongside its capacity to enhance growth performance [14,15]. Previous studies have suggested that sanguinarine-containing preparations can improve nutrient utilization, regulate immune responses, and support intestinal health in livestock and poultry [16,17]. However, the biological effects of sanguinarine may vary depending on dose, animal species, intestinal microbial composition, and developmental or production stage. More importantly, although sanguinarine has been considered a promising antibiotic alternative, its mechanism in yellow-feather broilers remains insufficiently defined. In particular, whether dietary sanguinarine improves growth performance through coordinated regulation of the intestinal barrier, immune-inflammatory balance, oxidative status, gut microbiota, and epithelial regenerative signaling has not been fully clarified.

The gut microbiota is increasingly recognized as a central mediator linking feed additives to intestinal function [18]. Beneficial bacteria generate SCFAs like butyrate, which supply energy to epithelial cells and are crucial for immune regulation, maintaining barrier integrity, and renewing epithelial cells [19,20]. Butyrate has the ability to activate G protein-coupled receptors such as GPR109A, thus contributing to anti-inflammatory responses and intestinal epithelial protection [21,22]. In parallel, Wnt/β-catenin signaling is a core pathway controlling intestinal epithelial proliferation, stem cell maintenance, and mucosal repair. Activation of Wnt3a, β-catenin, LRP5, and LRP6 is essential for restoring epithelial renewal after inflammatory injury [23]. Therefore, a microbiota–butyrate–GPR109A–Wnt/β-catenin signaling axis may represent a plausible mechanism by which dietary sanguinarine improves intestinal health. However, direct evidence linking sanguinarine-induced microbial remodeling to butyrate production, GPR109A activation, and epithelial repair remains limited.

To address this knowledge gap, the present study investigated the effects of dietary sanguinarine supplementation on growth performance, intestinal barrier function, immune and inflammatory responses, antioxidant status, cecal microbiota composition, predicted microbial functions, SCFA production, and GPR109A-associated Wnt/β-catenin signaling in yellow-feathered broilers. We further established an LPS-challenged intestinal organoid model to evaluate the potential direct effects of sanguinarine on epithelial recovery under inflammatory conditions. By integrating in vivo physiological and microbiome analyses with an in vitro intestinal organoid model, this study aimed to characterize the potential relationship among dietary sanguinarine, microbiota-associated metabolites, and epithelial homeostasis.

## 2. Materials and Methods

All experimental procedures and animal handling were approved by the Animal Care and Use Committee of Zhejiang University (Hangzhou, China; approval No. ZJU20160416) and were conducted in accordance with the guidelines of the committee. The sanguinarine (SA, purity ≥ 98%) used in this experiment was provided by Hunan Micolta Bioresource Co., Ltd. (Changsha, Hunan, China).

### 2.1. Experimental Design and Management

A total of 720 Lingnan yellow broilers were procured from the ZhengDa Broilers Development Center at Zhejiang University (Hangzhou, China) and subsequently reared at XinXing Broiler Farm (Jiaxing, China). The day-old chicks, with comparable weights, were randomly divided into four treatment groups, each consisting of six replicates of 30 birds. The treatments were as follows: (1) Control group (CTR): broilers fed a basal diet; (2) antibiotics group (ANTI): broilers fed the basal diet plus 250 mg/kg nosiheptide and 200 mg/kg oxytetracycline calcium; (3) SAL group: broilers fed a basal diet plus sanguinarine (0.5 mg/kg feed); (4) SAH group: broilers fed a basal diet plus sanguinarine (0.7 mg/kg feed).

The basal diet (Table 1) was formulated as mash following the Feeding Standard of Chicken (NY/T33-2004), promulgated by the Ministry of Agriculture of the PRC (PRC MoA, 2004) [24]. Broilers were housed under continuous 24 h lighting. The ambient temperature was initially maintained at 32 °C and gradually reduced by approximately 2 °C per week until it reached 24 °C. The feeding trial lasted for 63 days.

### 2.2. Slaughter and Sample Acquisition

At 63 days of age, the birds were individually weighed and identified before being euthanized by electrical stunning followed by exsanguination. Blood samples were collected and centrifuged to separate the serum samples. The intestine was gathered and washed with cold, sterile PBS simultaneously to eliminate any attached impurities, and the mucosa of the jejunum and ileum is gently scraped. The content samples of the cecum were gathered and preserved in liquid nitrogen until needed.

### 2.3. Analyses of Serum Biochemical Indices

Immunoglobulin concentrations in serum (IgG, IgM, IgA) and the capacity for total antioxidant activity (T-AOC) and MDA content, along with the activities of total SOD (T-SOD), glutathione peroxidase (GSH-Px), catalase (CAT), D-lactate acid (D-LA), diamine oxidase (DAO) and myeloperoxidase (MPO) were evaluated using assay kits (Nanjing Jiancheng, Bioengineering Institute, Nanjing, China).

### 2.4. RNA Extraction and RT-qPCR

Total RNA was extracted using the RNAiso Plus Kit (Takara, Kusatsu, Shiga, Japan) in accordance with the manufacturer’s instructions. Purity and concentration of the total RNA were determined using a NanoDrop spectrophotometer (ND-2000, Thermo Fisher Scientific, Wilmington, DE, USA). Reverse transcription of total RNA was performed with a M-MLV reverse transcriptase kit following the manufacturer’s protocol. Quantitative RT-PCR detection was carried out on an ABI 7500 Fast Real-Time PCR System using SYBR Green (Takara, Kusatsu, Shiga, Japan, SYBR Premix Ex Taq II Kit). Each sample was run in triplicate, and the primers are listed in Table S2. Relative expression levels were calculated using the 2^−ΔΔCt^ method, with *β-actin* serving as the internal reference gene.

### 2.5. Intestinal Microbial DNA Extraction and High-Throughput Sequencing

Microbial genomic DNA was extracted from the cecal contents of 63-day-old broilers under sterile conditions using the TIANamp Stool DNA Kit (Tiangen, Beijing, China) according to the manufacturer’s instructions. The V3–V4 region of the bacterial 16S rRNA gene was amplified using the 341F/805R primer pair, and paired-end sequencing was performed on an Illumina HiSeq 2500 platform. For the 18 samples included in the microbiota analysis, a total of 1,118,097 raw reads were generated. After quality control and read processing, 1,110,214 clean reads and 994,315 effective reads were retained, corresponding to an average of 61,679 clean reads and 55,240 effective reads per sample. The minimum number of effective reads retained for an individual sample was 36,453. The sequencing data were reanalyzed using QIIME 2 (v2026.7) with an ASV-based workflow. After demultiplexing, quality filtering, denoising, paired-end read merging, and chimera removal, amplicon sequence variants (ASVs) were inferred using the DADA2 algorithm without clustering at a predefined sequence-similarity threshold. Taxonomic assignment of representative ASV sequences was performed using the q2-feature-classifier plugin with a Naïve Bayes classifier trained against the SILVA 138.1 reference database. Sequences assigned to mitochondria, chloroplasts, or non-bacterial taxa were removed before downstream analyses. Alpha diversity was assessed using the Shannon, Simpson, ACE, Chao1, and Good’s coverage indices. Rarefaction curves were generated to evaluate sequencing depth and sampling completeness. Beta diversity was evaluated using unweighted UniFrac distances. Principal coordinate analysis (PCoA) and nonmetric multidimensional scaling (NMDS) were used to visualize differences in microbial community structure among treatment groups.

Statistical differences in overall microbial community composition were evaluated by permutational multivariate analysis of variance (PERMANOVA) using the adonis2 function in the vegan package in R (4.6.1), based on the unweighted UniFrac distance matrix with 999 permutations. PCoA and NMDS were used solely for visualization and were not interpreted as statistical tests of group differences. Taxonomic composition was summarized at the phylum, class, order, family, and genus levels. Differentially abundant taxa among groups were identified using linear discriminant analysis effect size (LEfSe), with a Kruskal–Wallis test threshold of *p* < 0.05 and an LDA score > 3.0. Taxa meeting both criteria were considered discriminatory features among treatment groups.

### 2.6. Detection of SCFA Levels

Previous reports [25] were used to prepare the sample. The concentrations of short-chain fatty acids (SCFAs) were measured on a gas chromatograph with the following conditions: carrier gas flow of 2 mL/min; an initial column temperature of 90 °C held for 6 min; a temperature ramp of 10 °C/min up to 200 °C followed by another 6 min hold. The instrument used was an Agilent 7890 GC (Palo Alto, CA, USA). The flame ionization detector (FID) operated at 250 °C with hydrogen as the fuel at 30 mL/min and air at 300 mL/min.

### 2.7. Intestinal Crypt Isolation and Organoid Establishment

The preparation of intestinal organoids was carried out in accordance with the methods that Ding et al. disclosed [26]. After the broiler jejunum had been dissected and fragmented, the supernatant was washed with DPBS twenty times to remove any remaining debris. Crypt fractions were separated by centrifuging at 290× *g* for 5 min. This followed vortexing of the intestinal pieces and filtration through a 70 µm cell strainer. Rudimentary crypts were formed by spinning at 200× *g* for 3 min to remove single cells. Equal volumes of Matrigel (BD Biosciences/Corning, NY, USA) and IntestiCult Organoid Growth Medium (STEMCELL Technologies, Vancouver, BC, Canada) were used to plate the crypts onto 24-well plates. The plates were incubated at 37 °C with 5% CO_2_ for 15 min. After the Matrigel had solidified, IntestiCult Organoid Growth Medium was applied. Organoid studies were treated as follows: CON (basic medium), SA (0.05 μM SA treatment for 24 h), LPS (200 μg/mL LPS treatment for 24 h), SA + LPS (0.05 μM SA pre-treatment for 24 h, then 200 μg/mL LPS treatment for 24 h). Jejunal organoid measurements were derived from the description cited earlier [27].

### 2.8. Statistical Analysis

SPSS Statistics (version 20.0; IBM Corp., Armonk, NY, USA) was used to perform a one-way ANOVA and subsequent multiple comparison tests in order to evaluate Tukey’s multiple comparison test. The mean values were reported with their respective standard deviation (SD), and *p* < 0.05 was considered to indicate a statistically significant difference. The graphing software utilized was GraphPad Prism 8.4.2.

## 3. Results

### 3.1. Sanguinarine Improved the Growth Performance in Broilers

Compared with the CTR group, both SAH and ANTI significantly increased BW at days 21 and 63 and ADG during days 1–21 and 42–63, while reducing F/G during days 21–42 and 42–63 (*p* < 0.05; Table 1). In addition, BW at day 42 was significantly higher in the ANTI group than in the SAH group (*p* < 0.05). For the remaining growth-performance parameters, no significant differences were observed between the ANTI and SAH groups (*p* > 0.05). SAL supplementation did not significantly affect growth performance compared with the CTR group.

### 3.2. Sanguinarine Improved the Intestinal Physical Barrier Function in Broilers

**Figure 1:**
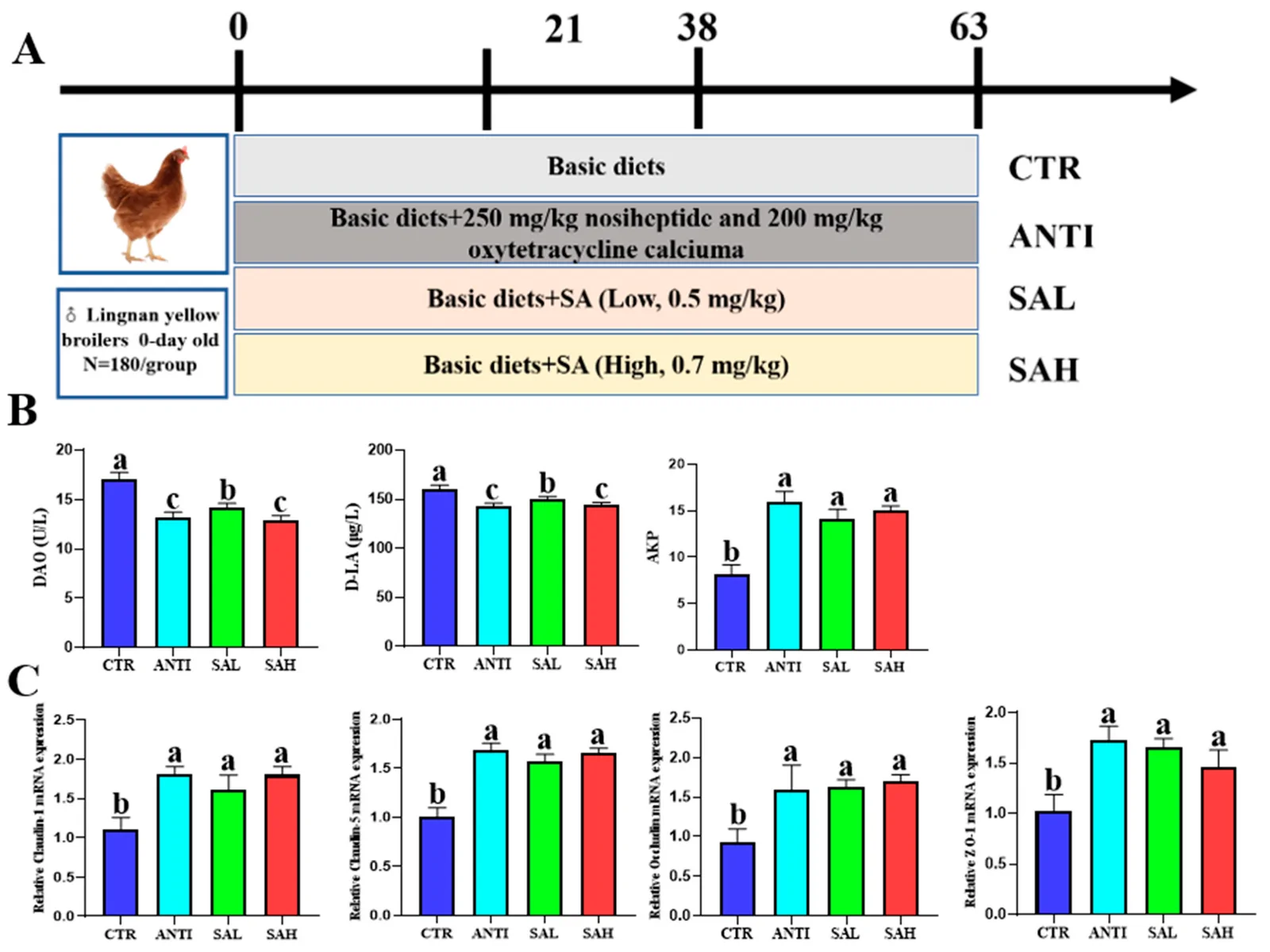
Effects of adding Sanguinarine to the diet on the intestinal physical barrier. (**A**) Overview of experimental design. (**B**) Activities of diamine oxidase (DAO), D-lactate (D-LA), and alkaline phosphatase (AKP) in serum (**C**) the relative mRNA expression level of *Claudin-1*, *Claudin-5*, *Occludin,* and *ZO-1*. Data are presented as means ± SD (*n* = 6). ZO-1, zonula occluden 1; ^a, b, c^ Means within a row with different superscripts are significantly different (*p* < 0.05).

Broilers in the ANTI and SAH groups had significantly lower DAO and D-LA levels than those in the CTR group. Additionally, SAH and ANTI groups markedly upregulated the expression of jejunal mucosal genes *Claudin-1*, *Claudin-5*, *Occludin*, and *ZO-1* (Figure 1B,C). There were no significant differences (*p* > 0.05) between the SAH and ANTI groups in DAO, D-LA contents, or in *Claudin-1*, *Occludin,* and ZO-1 gene expression. In conclusion, dietary supplementation with SAH improved intestinal barrier function in broilers in a dose-dependent manner.

### 3.3. Sanguinarine Enhanced the Antioxidant Status in Broilers

Analysis of antioxidant parameters in the serum and jejunal mucosa showed that T-AOC levels and T-SOD activities were significantly higher in the SAH and ANTI groups than in the CTR group (*p* < 0.05; Table 2). In addition, SAH and ANTI supplementation significantly reduced serum MDA levels compared with the CTR group (*p* < 0.05). No significant differences in GSH-Px or CAT activity were observed among the groups (*p* > 0.05).

### 3.4. Sanguinarine Enhanced Immune Function and Reduced Inflammatory Responses in Broilers

**Figure 2:**
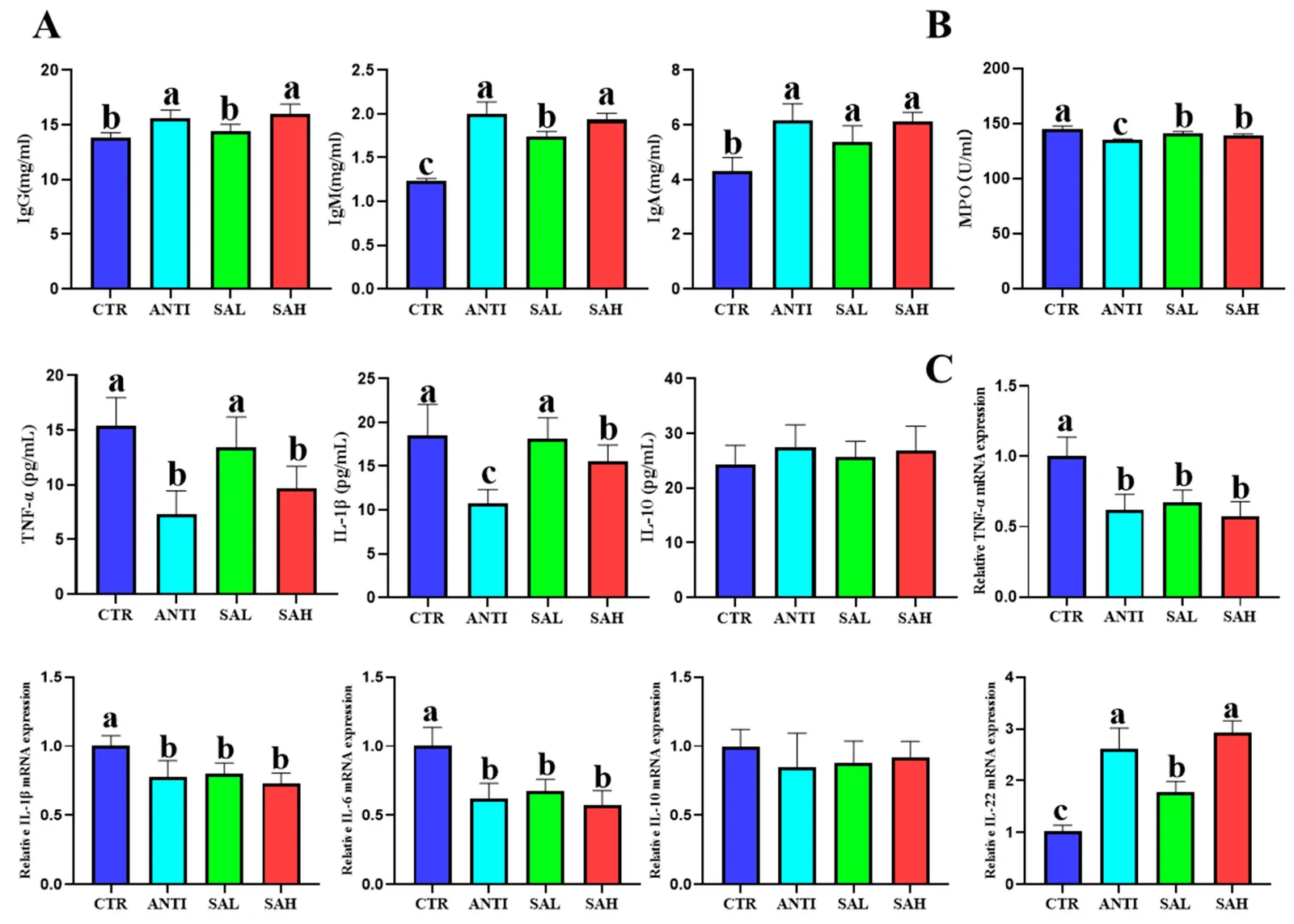
Dietary Sanguinarine enhanced immune function and regulated inflammatory responses in broilers. (**A**) IgG, IgM, and IgA content in serum. (**B**) MPO activity, TNF-α, IL-1β, and IL-10 content in serum. (**C**) Relative mRNA expression of *TNF-α*, *IL-1β*, *IL-6*, *IL-10*, and *IL-22*. Different lowercase letters indicate significant differences among groups. Data are presented as means ± SD (*n* = 6); ^a, b, c^ Means within a row with different superscripts differ significantly (*p* < 0.05).

The serum levels of IgG, IgM and IgA in ANTI, SAL and SAH were significantly higher than those in CTR. Serum IgM and IgG concentrations were significantly higher in the SAH and ANTI groups than in the SAL group (*p* < 0.05), whereas no significant differences (*p* > 0.05) were observed in the other immunoglobulin indices among the treatment groups (Figure 2A). SAH supplementation also significantly decreased the concentration of LPS and MPO compared with the control group (Figure 2B). There were no obviously (*p* > 0.05) differences in the levels of IgG, IgG and LPS between the SAH and ANTI groups. Figure 2C showed that the SAH and ANTI groups notably (*p* < 0.05) increased the *IL-10* and *IL-22* gene expression levels and downregulated the IL-1β, IL-6, and TNF-α gene expression.

### 3.5. Cecum Microbiota Analysis

#### 3.5.1. Microbiota Diversity in Intestinal Contents

**Figure 3:**
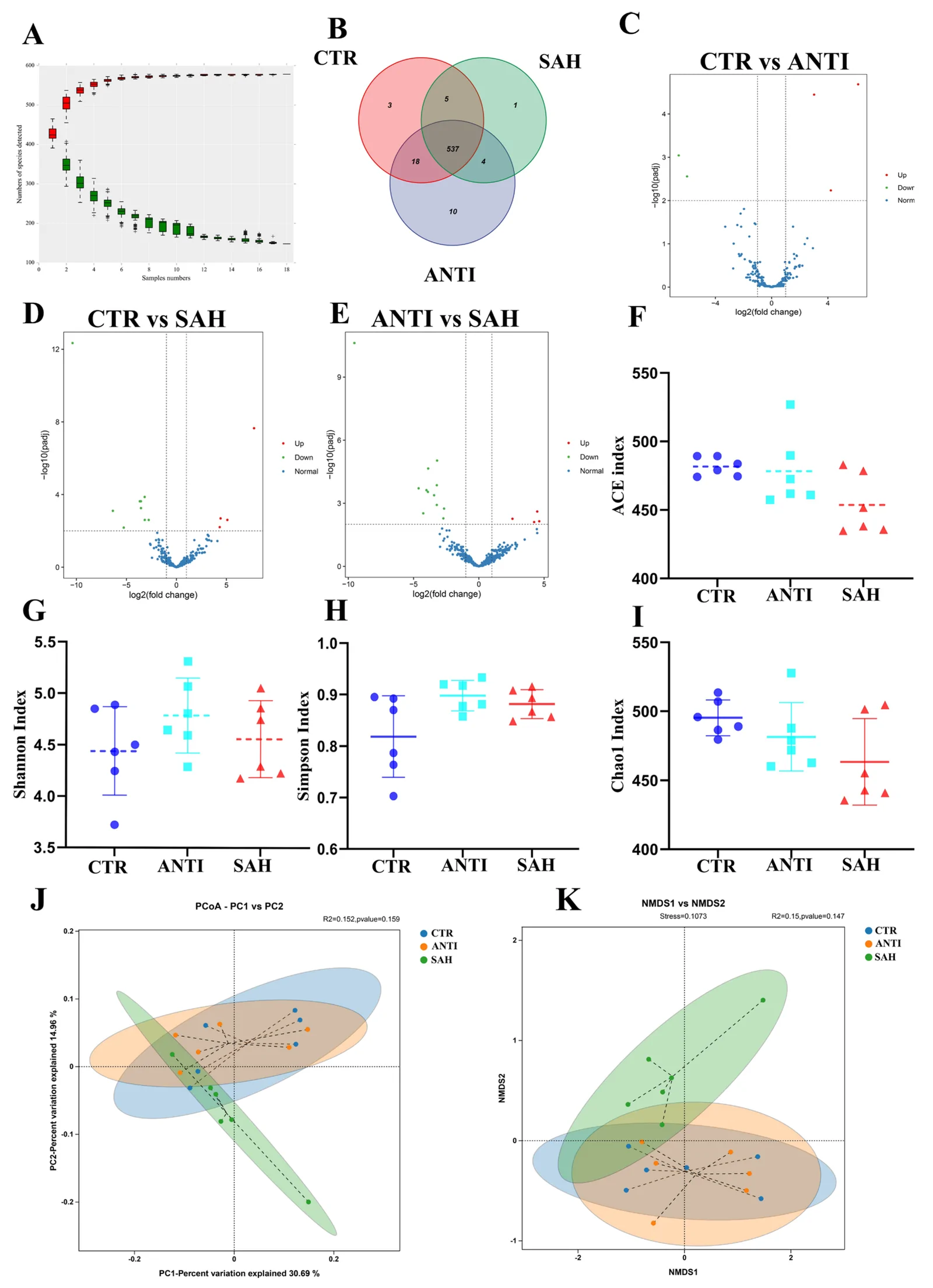
Sanguinarine improved cecal microbiota diversity in broilers based on ASV level. (**A**) Species Cumulative Curve. (**B**) Venn diagram; (**C**–**E**) Volcano plots among all groups; (**F**) ACE index, (**G**) Shannon index, (**H**) Simpson index, (**I**) Chao1 index, (**J**) PCoA of weighted Unifrac distance, (**K**) NMDS analysis of unweighted Unifrac distance.

Prior to downstream microbial community analysis, sequencing data were subjected to quality control and ASV-based processing. Rarefaction curves approached a plateau, indicating that the sequencing depth was sufficient to capture most of the bacterial diversity present in the samples (Figure 3A). Venn analysis identified 537 ASVs shared across all experimental groups. In addition, 31 ASVs were shared between the CTR and ANTI groups, whereas 542 and 541 ASVs were shared between the CTR and SAH groups and between the ANTI and SAH groups, respectively (Figure 3B–E).

Alpha-diversity analysis showed no significant differences in microbial richness or diversity among the treatment groups, as assessed by the Shannon, Simpson, ACE, and Chao1 indices (*p* > 0.05; Figure 3F–I). PCoA and NMDS showed partially distinct clustering patterns among the CTR, ANTI, and SAH groups (Figure 3J,K). However, formal statistical testing did not demonstrate a significant overall difference in microbial community composition among the groups. For the PCoA-based comparison, PERMANOVA yielded R^2^ = 0.152 and *p* = 0.159, whereas the corresponding analysis associated with the NMDS ordination yielded R^2^ = 0.150 and *p* = 0.147. The NMDS model showed an acceptable goodness of fit (stress = 0.1073). Thus, although the ordination plots suggested some treatment-associated separation, the overall differences in beta diversity did not reach statistical significance.

#### 3.5.2. Overall Structure Modulation of Gut Microbiota

**Figure 4:**
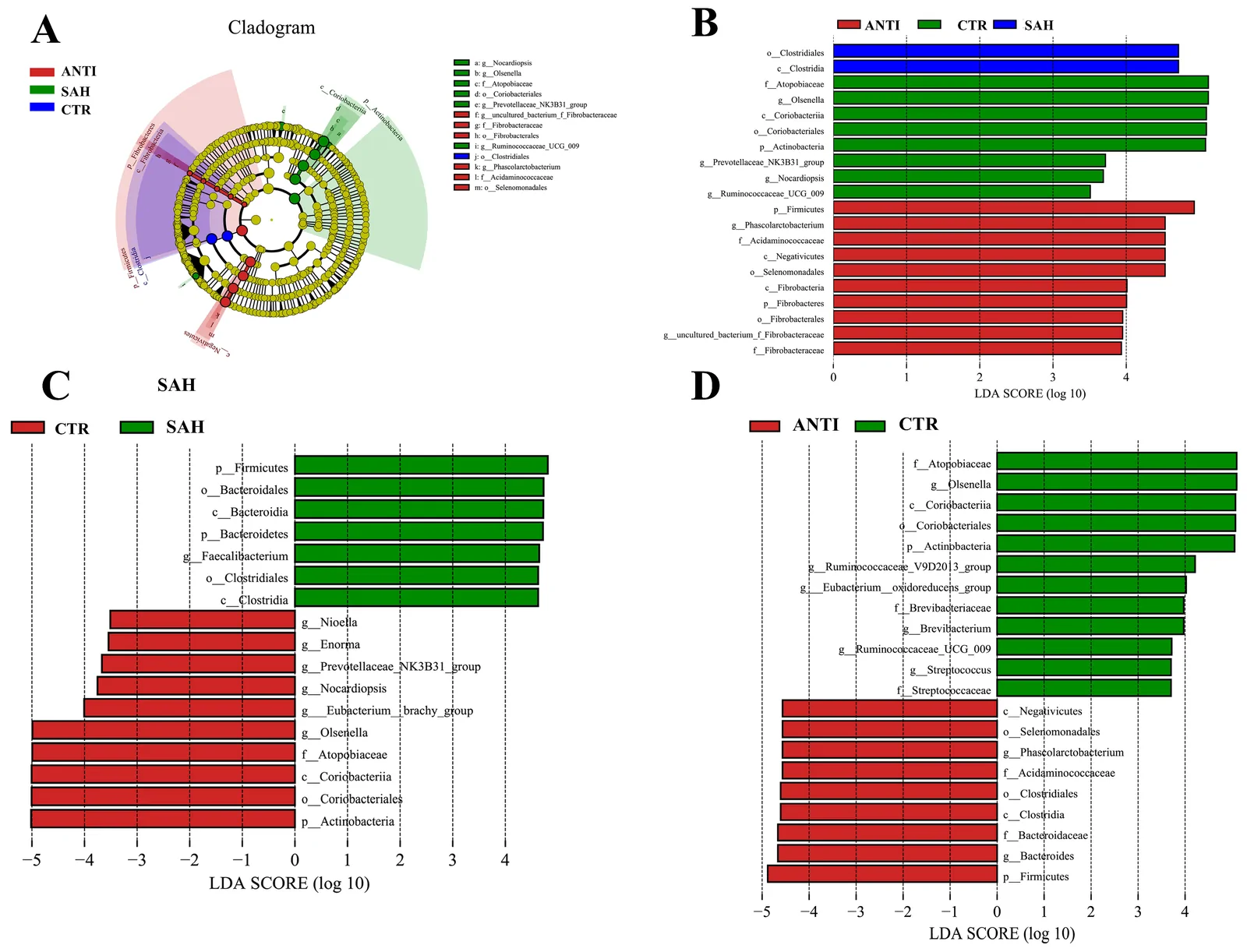
LEfSe cladogram and LEfSe bar. (**A**) cladogram diagram among all groups. (**B**) LDA score diagram among all group. (**C**) CTR vs. SAH, (**D**) CTR vs. ANTI.

Consistent with these community-level differences, LEfSe analysis (Kruskal–Wallis *p* < 0.05 and LDA score > 3.0) identified distinct discriminatory bacterial taxa among the experimental groups (Figure 4). At the genus level, SAH supplementation was associated with an increased relative abundance of Faecalibacterium and decreased relative abundances of *Olsenella*, *Enorma, Nioella,* and *Prevotellaceae_NK3B31_group*. In contrast, the ANTI group exhibited increased relative abundances of *Olsenella, Brevibacterium,* and *Ruminococcaceae_UCG-009*, together with decreased relative abundances of *Phascolarctobacterium* and *Bacteroides*.

#### 3.5.3. Predicted Metabolic Functions in the Gut Microbiota

**Figure 5:**
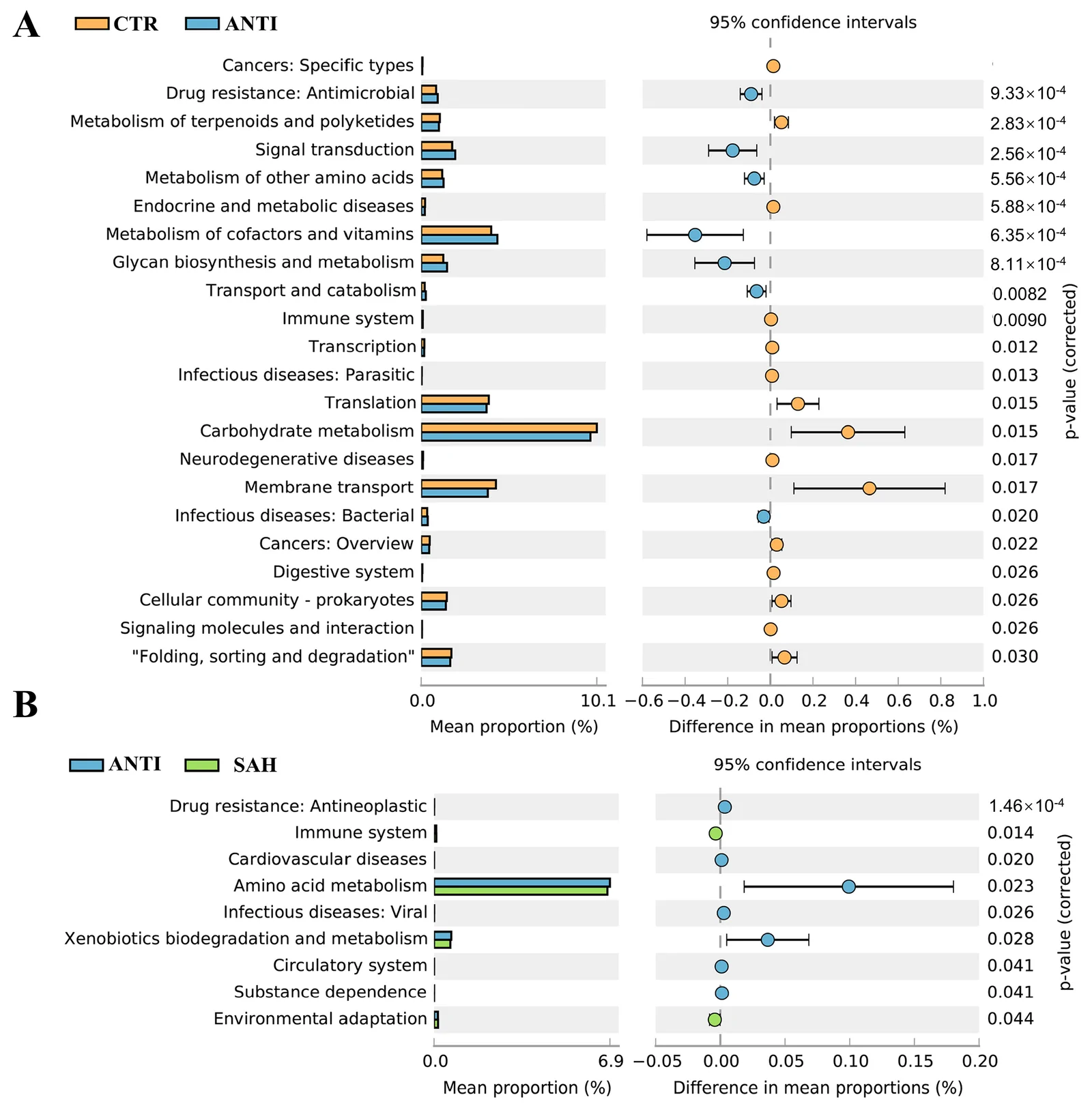
Comparison to predicted metabolic pathway abundance between the groups by statistical analysis of taxonomic and functional profiles (STAMP) at Level 2. (**A**) CTR vs. ANTI, (**B**) CTR vs. SAH.

PICRUSt-based functional prediction was performed to investigate potential changes in microbial metabolic functions following SAH and ANTI supplementation (Figure 5). Functional prediction analysis revealed that both SAH and ANTI interventions enriched microbial pathways associated with glycan biosynthesis and metabolism, cofactor and vitamin metabolism, as well as the metabolism of other amino acids (Figure 5A,B). Furthermore, SAH significantly increased the abundance of microbiota related to the immune system and environmental adaptation.

### 3.6. Correlation Heat Map

**Figure 6:**
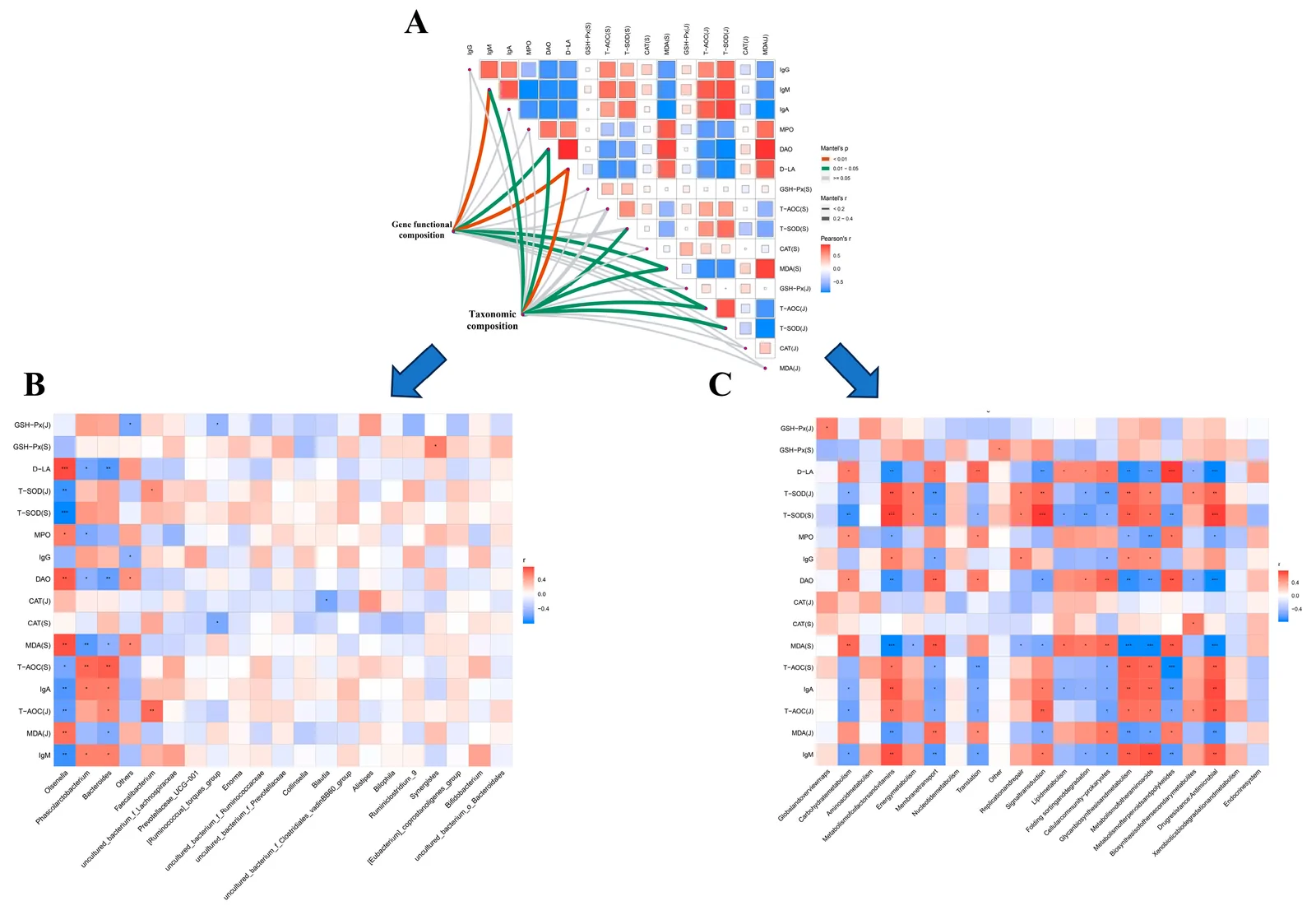
Integrative analysis linking phenotypic traits, bacterial communities, and predicted metabolic pathways. (**A**) Interrelationships between host phenotypic parameters, where the color spectrum indicates the strength of the correlation coefficient. Combined taxonomic and functional profiles were fitted against each phenotype via Mantel tests (utilizing Bray–Curtis dissimilarity metrics); line thickness (edge width) reflects Mantel’s *r* statistic, and line color designates statistical significance. (**B**) Heatmap profiling the correlations between phenotypic metrics and bacterial abundances. (**C**) Correlation matrix between phenotypic parameters and predicted microbial metabolic functions. Both color shading and circle dimensions correspond to the magnitude of the correlation coefficients. * *p* < 0.05, ** *p* < 0.01, *** *p* < 0.001.

To further elucidate the specific relationships between phenotypic parameters and either microbial functional structures or metabolic profiles, Spearman correlation analysis was employed (Figure 6A). As illustrated in Figure 6B, Spearman correlation analysis revealed that pro-inflammatory markers (MPO and NO), the oxidative stress indicator (MDA), and intestinal permeability indices (D-LA and DAO) were positively associated with *Olsenella* abundance, whereas they exhibited inverse relationships with *Phascolarctobacterium*. Furthermore, serum immunoglobulins (IgG, IgM, and IgA) along with antioxidant parameters (T-AOC and T-SOD) exhibited significant positive correlations with the relative abundances of *Phascolarctobacterium* and *Faecalibacterium*. As illustrated in Figure 6C, the correlation matrix revealed that serum immunoglobulins and antioxidant parameters were positively associated with pathways including glycan biosynthesis/metabolism, signal transduction, and the metabolism of cofactors, vitamins, and other amino acids. Conversely, these host physiological indices exhibited inverse relationships with carbohydrate metabolism, translation, membrane transport, cellular community-prokaryotes, and folding, sorting and degradation. These results indicated that the promoted intestinal health of broiler chicks might be relevant to the altered structure of the cecal microbiota manipulated by SA supplementation.

### 3.7. Sanguinarine Regulated SCFA Production and Activated GPR109A-Related Wnt/β-Catenin Signaling Pathway

**Figure 7:**
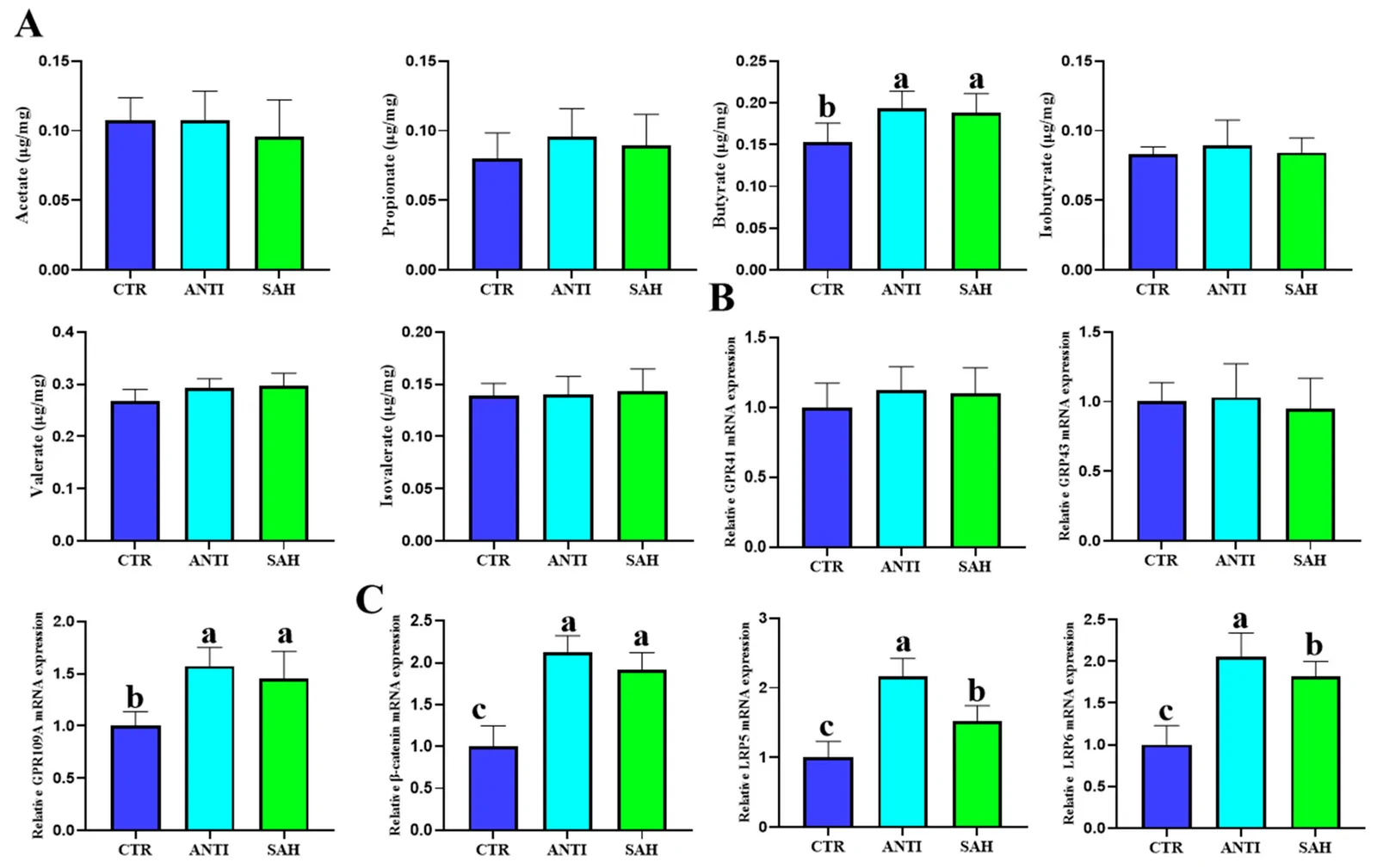
Modulation of butyrate biosynthesis, GPR109A transcription, and Wnt/β-catenin cascades by dietary SA. (**A**) Profile of cecal short-chain fatty acids (SCFAs), comprising acetate, propionate, butyrate, isobutyrate, valerate, and isovalerate. (**B**) Relative transcript levels of SCFA-sensing receptors (GPR41, GPR43, and GPR109A). (**C**) Relative mRNA abundances of key components in the Wnt/β-catenin signaling cascade (β-catenin, LRP5, and LRP6). Columns designated with distinct lowercase superscripts indicate statistically significant differences (*p* < 0.05). Values are expressed as means ± SD (*n* = 6).

Compared with the CTR group, butyrate was significantly increased in the ANTI and SAH groups, whereas acetate, propionate, isobutyrate, valerate, and isovalerate showed no obvious significant differences among groups (Figure 7A). These results suggested that butyrate may be the major SCFA responsive to dietary intervention in this model. The expression of SCFA receptors was further assessed. GPR41 and GPR43 showed no marked intergroup differences, whereas GPR109A expression was significantly increased in the ANTI and SAH groups (Figure 7B). Since GPR109A is an important receptor involved in butyrate sensing and intestinal epithelial regulation, these findings suggested that SA may enhance butyrate-associated host signaling mainly through GPR109A. In addition, the mRNA level of Wnt/β-catenin pathway (β-catenin, LRP5, and LRP6) was increased after intervention, although the degree of activation differed between ANTI and SAH groups (Figure 7C). These results suggested that SA may promote intestinal epithelial renewal by activating butyrate/GPR109A-related Wnt/β-catenin signaling.

### 3.8. Sanguinarine Protected Intestinal Organoids Against LPS-Induced Growth Impairment

**Figure 8:**
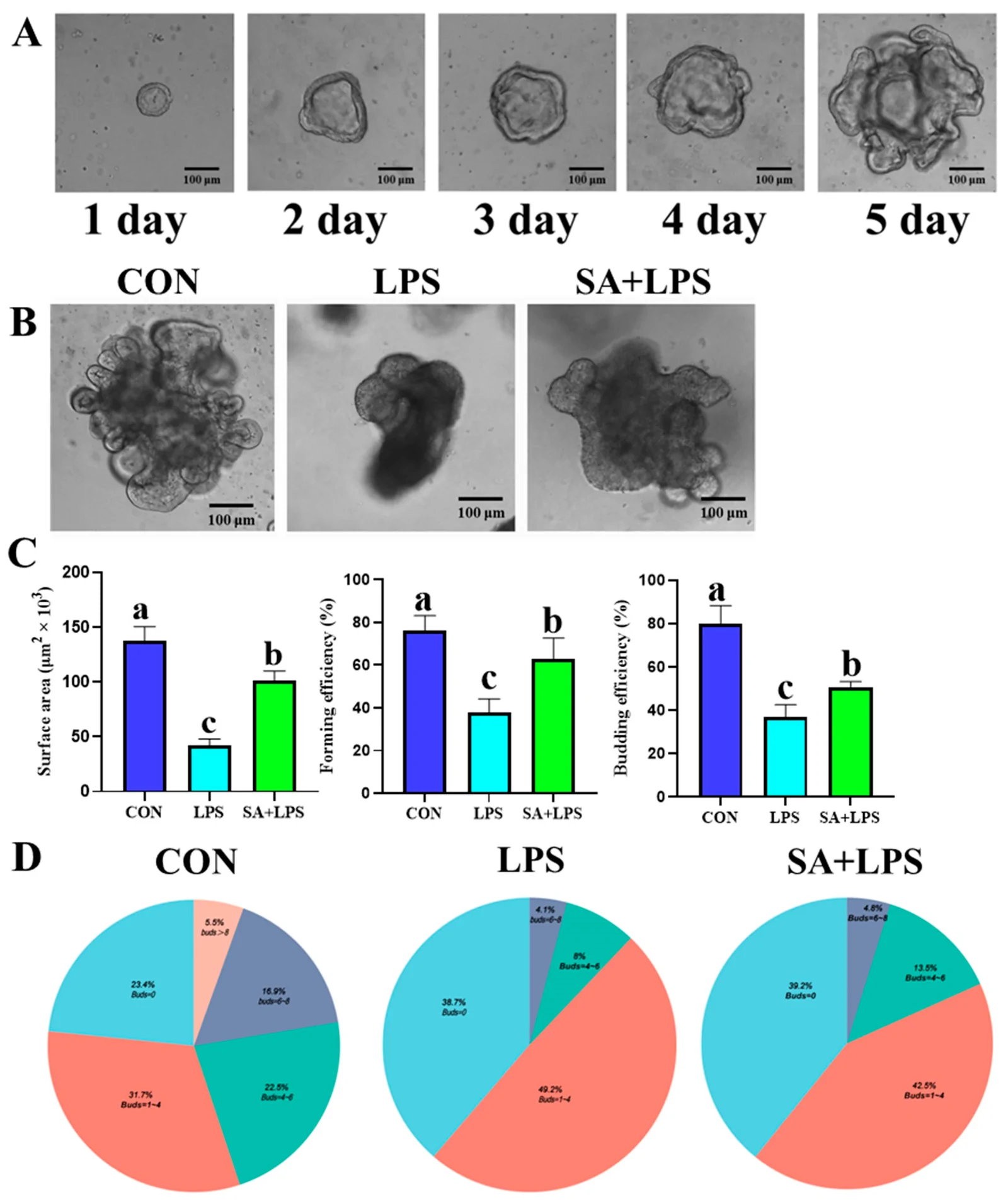
Protective efficacy of sanguinarine against LPS-induced growth suppression in intestinal organoids. (**A**) Time-course micrographs displaying organoid development from day 1 through day 5. (**B**) Morphological profiles of organoids across the CON, LPS, and SA + LPS cohorts. (**C**) Quantitative evaluation of organoid surface area, organoid-forming efficiency, and budding efficiency. (**D**) Proportional distribution of organoid developmental grades within each treatment group. Scale bars are specified within the respective panels. Bars assigned with distinct lowercase superscripts indicate statistically significant differences (*p* < 0.05). Data are expressed as means ± SD (*n* = 6).

Intestinal organoids were used to examine the effects of sanguinarine on epithelial recovery under inflammatory stress. During culture, organoids gradually expanded from day 1 to day 5, confirming successful establishment of the intestinal organoid model (Figure 8A). LPS stimulation markedly impaired organoid growth, as shown by reduced organoid size, decreased budding, and poorer morphological development. In contrast, SA treatment partially rescued LPS-induced organoid injury, with larger organoids and improved budding morphology compared with the LPS group (Figure 8B). RT-qPCR analysis showed that LPS significantly reduced organoid surface area, forming efficiency, and budding efficiency, whereas SA treatment increased these parameters compared with LPS stimulation alone (Figure 8C). The organoid grading distribution further showed that LPS increased the proportion of poorly developed organoids, while SA treatment shifted the distribution toward better-developed organoids with improved budding capacity (Figure 8D). These results indicated that sanguinarine protected intestinal organoids against LPS-induced growth inhibition and promoted epithelial regenerative capacity.

### 3.9. Sanguinarine Restored Organoid Proliferation and Wnt/β-Catenin Signaling Under LPS Stimulation

**Figure 9:**
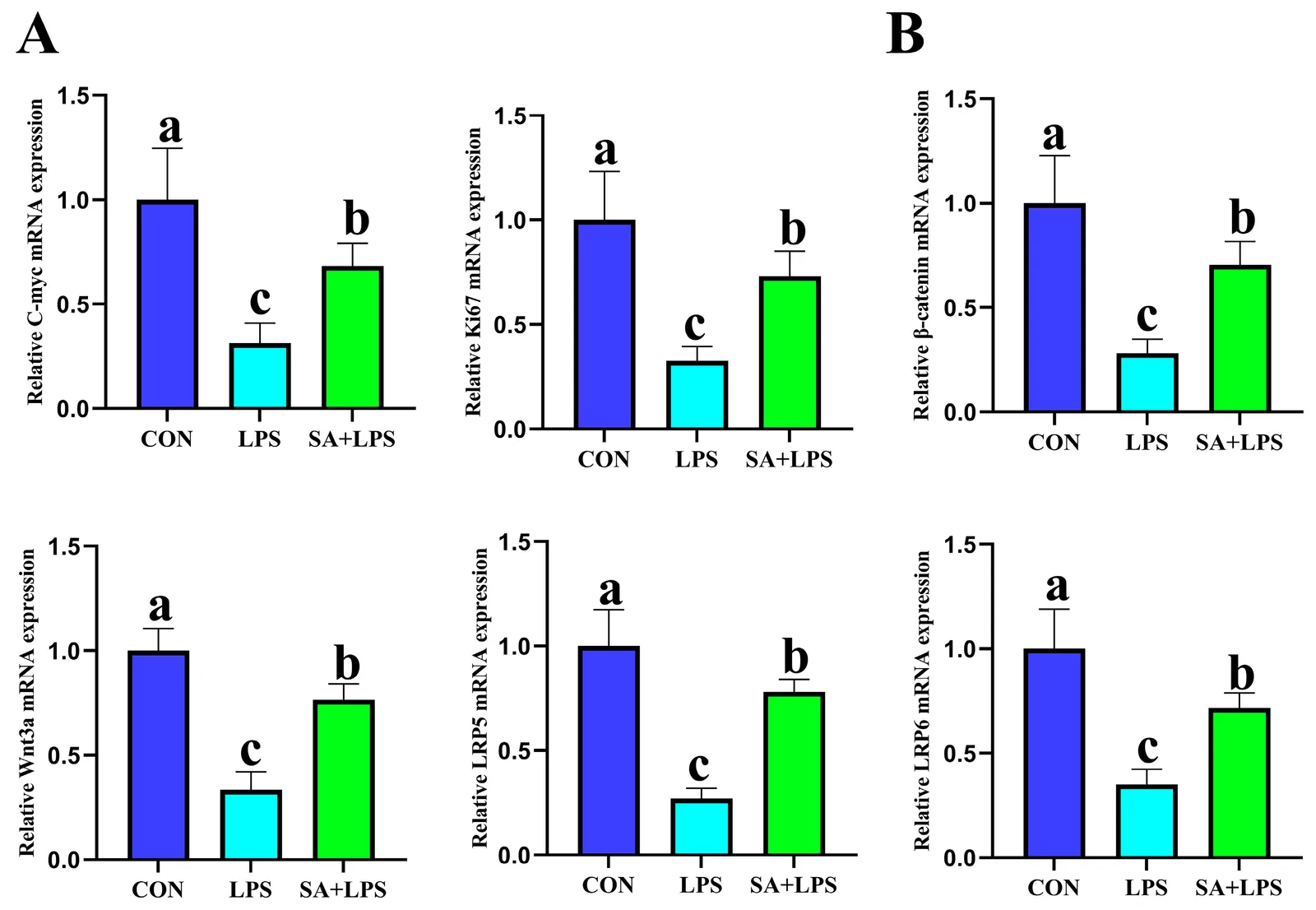
SA restored organoid proliferation and Wnt/β-catenin signaling under LPS stimulation. (**A**) Relative mRNA expression of proliferation-related genes, including C-myc and Ki67. (**B**) Relative mRNA abundances of core components within the Wnt/*β*-catenin signaling cascade, specifically *β-catenin*, *Wnt3a*, *LRP5*, and *LRP6*. Spatial scale dimensions are specified within the respective panels. Bars assigned with unshared lowercase letters indicate statistically significant variations among experimental groups (*p* < 0.05). Values are expressed as means ± SD (*n* = 6).

Gene expression analysis further showed that LPS suppressed the mRNA expression of proliferation-related genes C-myc and Ki67, whereas SA treatment significantly restored their expression (Figure 9A). In addition, LPS downregulated Wnt/β-catenin pathway-related genes, including β-catenin, Wnt3a, LRP5, and LRP6. SA treatment reversed these inhibitory effects and partially reactivated Wnt/β-catenin signaling (Figure 9B). These results suggested that sanguinarine promoted epithelial repair under inflammatory stress by restoring proliferation, and activating Wnt/β-catenin-mediated regenerative signaling.

## 4. Discussion

Our findings show that dietary sanguinarine supplementation improved growth performance and intestinal health in yellow-feathered broilers. The main findings were as follows: high-dose sanguinarine supplementation increased body weight and average daily gain and reduced the feed-to-gain ratio; sanguinarine enhanced intestinal barrier function, as reflected by decreased serum DAO, D-LA, and LPS levels and increased expression of the barrier-related genes *Claudin-1*, *Claudin-5*, *Occludin*, *ZO-1*, and *MUC2*; sanguinarine improved antioxidant and immune status by increasing T-AOC, T-SOD, IgG, IgM, and IgA while reducing MDA, MPO, and pro-inflammatory cytokines; sanguinarine reshaped the cecal microbial community, increased beneficial bacterial taxa such as *Faecalibacterium* and *Phascolarctobacterium*, and reduced potentially unfavorable taxa such as *Olsenella*, *Prevotellaceae_NK3B31_group*, *Enorma*, and *Nioella*. Sanguinarine was also associated with increased butyrate production, enhanced GPR109A expression, and upregulation of Wnt/β-catenin-related genes. Furthermore, in vitro organoid experiments showed that sanguinarine protected intestinal organoids against LPS-induced injury by restoring Ki67 and *C-myc* expression, and increasing the expression of genes related to Wnt/β-catenin signaling. Collectively, these findings suggest that sanguinarine may improve intestinal health through coordinated modulation of the gut microbiota, butyrate production, GPR109A expression, and Wnt/β-catenin-related signaling.

Historically, antibiotic growth promoters have been widely used to improve feed efficiency and reduce enteric disorders in poultry production [28].Nevertheless, rising concerns regarding antimicrobial resistance and food safety have accelerated the search for phytogenic alternatives [29]. Previous studies have reported that sanguinarine or sanguinarine-containing phytobiotics can enhance the growth performance of broiler chickens, modulate intestinal microbiota, and support intestinal health, which is consistent with our findings [15,30]. In the present study, SAH significantly improved BW and ADG and reduced F during several feeding periods, and its effects on several growth-performance parameters were comparable to those observed with antibiotic supplementation. These findings suggest that dietary sanguinarine may represent a potential alternative to antibiotic growth promoters in yellow-feathered broiler production. Notably, SAL did not significantly improve growth performance, suggesting that the effects of sanguinarine may vary with dietary dose.

This dose-dependent pattern is consistent with the biological characteristics of plant-derived alkaloids, whose effects may depend on exposure level, intestinal microbial context, and host physiological state [31]. The improvement in growth performance may be closely linked to enhanced intestinal barrier integrity [32]. The intestinal barrier is essential for nutrient absorption and host defense, and its impairment can increase antigen entry, inflammation, and energy loss [33]. DAO and D-LA are commonly used as indicators of intestinal mucosal damage and permeability, while tight junction proteins such as Claudin, Occludin, and ZO-1 maintain epithelial paracellular integrity [34,35]. In poultry, intestinal permeability and tight junction stability are increasingly recognized as practical biomarkers of gut health [36,37]. In the present study, SAH reduced DAO and D-LA levels and upregulated *Claudin-1*, *Claudin-5*, *Occludin*, *ZO-1*, and *MUC2* expression. These findings suggest that sanguinarine strengthened epithelial barrier integrity and mucus-associated defense. Previous broiler studies also reported that phytogenic compounds and sanguinarine-containing additives can enhance gut barrier function and intestinal morphology, supporting the present conclusion [38].

Inflammatory responses and oxidative stress are two important pathological processes that impair intestinal epithelial homeostasis [39]. Excessive inflammatory activation can damage tight junctions and disturb nutrient absorption, whereas oxidative stress can induce lipid peroxidation and epithelial dysfunction [40]. SA has been reported to exert anti-inflammatory and antioxidant effects in multiple experimental models [41,42]. In the present study, SAH decreased MPO and LPS levels and downregulated IL-1β, IL-6, and TNF-α expression, while increasing IL-10 and IL-22 expression. Meanwhile, SAH increased T-AOC and T-SOD and reduced MDA. These findings indicate that sanguinarine attenuated pro-inflammatory responses and improved several indicators of antioxidant status. However, GSH-Px and CAT activities were not significantly altered. Thus, the antioxidant effects of sanguinarine did not involve a generalized enhancement of all endogenous antioxidant enzymes. Instead, its effects appeared to be selective, particularly with respect to reducing lipid peroxidation and improving specific antioxidant parameters. Accordingly, the antioxidant action of sanguinarine should be interpreted as selective modulation of redox homeostasis rather than broad activation of the entire antioxidant defense system.

The immune regulatory effect of sanguinarine was also evident [43]. Serum immunoglobulin concentrations, including IgG, IgM, and IgA, were increased after sanguinarine supplementation, indicating enhanced humoral immune support. IgA is particularly important for mucosal defense because it participates in immune exclusion of luminal antigens and helps maintain microbial balance [44]. The concomitant increase in *IL-10* and *IL-22* further suggests that sanguinarine may favor a more anti-inflammatory intestinal immune environment. This is relevant to intestinal health because uncontrolled inflammatory activation often compromises epithelial integrity, whereas appropriate immune regulation supports barrier repair and microbial tolerance. Thus, the improved immune status observed in this study may partly contribute to the reduced permeability markers and improved growth performance observed in SAH-treated broilers.

The gut microbiota is a central mediator of feed additive effects [45]. In this study, alpha diversity indices did not differ significantly among groups, suggesting that sanguinarine did not markedly change overall microbial richness or evenness. However, PCoA and NMDS analyses showed clear separation among treatments, indicating that sanguinarine reshaped the microbial community structure. This distinction is noteworthy because the principal microbiota response to sanguinarine appeared to involve compositional remodeling rather than a general increase in microbial diversity. At the genus level, SAH increased *Faecalibacterium* and reduced *Olsenella*, *Prevotellaceae-NK3B31_group*, *Enorma*, and *Nioella*. *Faecalibacterium* is widely recognized as a beneficial, anti-inflammatory, butyrate-associated bacterial taxon in intestinal ecosystems, and its increase is consistent with the observed improvement in butyrate production and improvement in inflammatory indices [46]. Correlation analysis further showed that inflammatory and barrier injury markers were positively associated with *Olsenella* but negatively associated with *Phascolarctobacterium*, whereas immunoglobulins and antioxidant enzymes were positively associated with *Phascolarctobacterium* and *Faecalibacterium*. These associations support a potential relationship between sanguinarine-induced microbial remodeling and improved intestinal physiology; however, they do not establish causality.

Among microbial metabolites, butyrate was the only metabolite that increased significantly following SAH supplementation [21]. SAH significantly increased butyrate, whereas acetate, propionate, isobutyrate, valerate, and isovalerate were not markedly changed. These findings suggest that butyrate may represent an important microbial metabolite associated with the intestinal effects of sanguinarine rather than reflecting a generalized increase in SCFA production. Butyrate is an important energy source for intestinal epithelial cells and regulates inflammation, epithelial barrier function, and immune homeostasis [47,48]. Moreover, butyrate can activate GPR109A, a receptor expressed in intestinal tissues and immune cells [22]. Previous studies have shown that GPR109A functions as a receptor for butyrate and niacin and contributes to anti-inflammatory responses and maintenance of intestinal homeostasis [49]. In this study, SAH significantly increased GPR109A expression, whereas GPR41 and GPR43 were not significantly altered. These results suggest that the increase in butyrate following sanguinarine supplementation was accompanied by selective enhancement of GPR109A expression rather than broad upregulation of SCFA receptors.

The activation of Wnt/β-catenin signaling provides a plausible epithelial repair mechanism downstream of the butyrate–GPR109A axis [50]. Wnt/β-catenin signaling is essential for intestinal epithelial renewal, crypt homeostasis, and stem/progenitor cell maintenance [51]. LRP5 and LRP6 are key co-receptors in canonical Wnt signaling and participate in intestinal epithelial development and regeneration [52]. In the current study, SAH increased the mRNA level of β-catenin, LRP5, and LRP6, indicating an association between sanguinarine supplementation and enhanced expression of Wnt/β-catenin pathway-related genes. This result is consistent with the improved barrier markers and reduced permeability indicators. However, the present in vivo data do not prove that GPR109A directly activates Wnt/β-catenin signaling. Rather, they support a potential mechanistic sequence in which increased butyrate production and enhanced GPR109A expression are accompanied by increased expression of Wnt/β-catenin-related genes. Future studies using GPR109A inhibition, receptor knockdown, or β-catenin pathway blockade are needed to verify this causal sequence.

The intestinal organoid experiments provided additional functional evidence for the epithelial protective effects of sanguinarine. LPS markedly impaired organoid growth, reduced surface area, formation efficiency, and budding efficiency, and increased the proportion of poorly developed organoids. Sanguinarine treatment partially reversed these changes, indicating a direct protective effect on intestinal epithelial cells that is independent of systemic physiological influences. At the transcriptional level, sanguinarine restored the expression of *C-myc*, *Ki67*, *β-catenin*, *Wnt3a*, *LRP5*, and *LRP6*. These findings suggest that sanguinarine attenuated epithelial apoptosis and supported proliferative recovery, accompanied by restoration of Wnt/β-catenin-related gene expression. Intestinal organoids are well-suited for studying epithelial-intrinsic repair mechanisms because they retain key features of intestinal epithelial growth and regeneration [53,54]. Therefore, the organoid data complement the in vivo findings and suggest that sanguinarine may exert direct effects on the intestinal epithelium in addition to modulating the gut microbiota.

Several limitations of this study should be acknowledged. First, although dietary sanguinarine supplementation was accompanied by alterations in gut microbial composition, increased butyrate concentrations, enhanced GPR109A expression, and changes in Wnt/β-catenin-related signaling, these findings remain largely associative and do not establish a complete causal microbiota–butyrate–GPR109A–Wnt/β-catenin pathway. Second, functional inference based on 16S rRNA gene sequencing relies on taxonomic composition and reference genomic information and does not directly measure microbial gene expression or metabolic activity. Therefore, the predicted functional changes should be interpreted cautiously and require further validation using approaches such as metagenomic, metatranscriptomic, or targeted functional analyses. Third, direct pathway-intervention experiments, including GPR109A inhibition or knockdown and pharmacological or genetic blockade of Wnt/β-catenin signaling, were not performed. Future studies incorporating these approaches will be necessary to determine the functional necessity of these signaling components and to establish the causal relationships within the proposed mechanism.

## 5. Conclusions

In conclusion, dietary sanguinarine supplementation improved growth performance and intestinal health in yellow-feathered broilers. These benefits were accompanied by enhanced intestinal barrier integrity, improved immune status, reduced inflammation and oxidative stress, selective remodeling of the cecal microbiota, increased butyrate production, and enhanced GPR109A and Wnt/β-catenin-related signaling. Intestinal organoid experiments further showed that sanguinarine attenuated LPS-induced epithelial apoptosis while promoting epithelial proliferation and regenerative signaling. Together, these findings support sanguinarine as a promising phytogenic feed additive for long-cycle yellow-feathered broiler production and extend current understanding of phytogenic feed additives by linking dietary sanguinarine supplementation with coordinated changes in gut microbiota, microbial butyrate production, and host intestinal signaling. The microbiota-associated butyrate/GPR109A/Wnt/β-catenin axis may therefore represent a plausible mechanism underlying the observed intestinal protective effects, although its causal role requires further experimental validation.

## Figures and Tables

**Table 1:** Effect of Sanguinarine on the growth performance of yellow feather broiler.

Stage	Indicator	CTR	ANTI	SAL	SAH	SEM	*p*-Value
BW	1 d	37.90	37.81	37.85	37.95	0.328	0.977
	21 d	413.51 ^b^	425.91 ^a^	418.45 ^ab^	428.86 ^a^	4.776	0.017
	42 d	1085.22 ^b^	1162.35 ^a^	1098.73 ^b^	1108.65 ^b^	13.709	0.001
	63 d	1934.80 ^c^	2065.88 ^a^	1961.31 ^c^	2007.67 ^b^	22.143	0.001
1–21 d	ADG	17.89 ^b^	18.48 ^a^	18.12 ^b^	18.61 ^a^	0.226	0.017
	ADFI	30.50 ^b^	31.38 ^a^	30.94 ^ab^	30.82 ^ab^	0.417	0.234
	F/G	1.71	1.70	1.71	1.67	0.029	0.296
21–42 d	ADG	31.99 ^b^	35.07 ^a^	32.39 ^b^	32.37 ^b^	0.664	0.001
	ADFI	80.79	82.27	79.88	80.16	1.370	0.332
	F/G	2.53 ^a^	2.35 ^b^	2.47 ^b^	2.48 ^b^	0.054	0.019
42–63 d	ADG	40.46 ^b^	43.02 ^a^	41.08 ^ab^	42.81 ^a^	1.054	0.06
	ADFI	100.09 ^a^	92.87 ^b^	104.46 ^a^	102.87 ^a^	2.172	0.001
	F/G	2.48 ^a^	2.16 ^b^	2.55 ^b^	2.41 ^b^	0.069	0.001

^a,b,c^ Means within a row with different superscripts differ significantly (*p* < 0.05). The experiment consisted of 6 replicates per treatment, with 30 broilers per replicate. BW, body weight; ADG, average daily gain; ADFI, average daily feed intake; F/G, feed-to-gain ratio.

**Table 2:** Serum and Jejunal mucosa antioxidant status of broiler ^1^.

Item	CTR	ANTI	SAL	SAH	SEM	*p*-Value
Serum						
T-AOC (U/mL)	6.67 ^b^	7.63 ^a^	6.98 ^b^	7.71 ^a^	0.256	0.001
T-SOD (U/mL)	129.17 ^b^	148.42 ^a^	144.32 ^a^	147.51 ^a^	4.204	0.001
CAT (U/mL)	7.28	7.37	7.08	7.56	0.248	0.314
GSH-Px (U/mL)	1405.22	1417.69	1408.86	1408.94	17.738	0.91
MDA (nmol/mL)	8.04 ^a^	6.98 ^c^	7.39 ^b^	7.00 ^c^	0.162	0.001
Jejunal mucosa						
T-AOC (U/mg prot)	2.69 ^c^	3.56 ^a^	3.20 ^b^	3.44 ^ab^	0.149	0.001
T-SOD (U/mg prot)	128.68 ^c^	152.25 ^a^	141.59 ^b^	153.21 ^a^	2.561	0.001
CAT (U/mg prot)	13.47	13.30	13.06	13.06	0.334	0.546
GSH-Px (U/mg prot)	2232.55	2258.62	2262.43	2246.94	35.928	0.839
MDA (U/mg prot)	3.47 ^a^	2.65 ^c^	2.98 ^b^	2.57 ^c^	0.131	0.001

^1^ Data represent the mean values derived from 6 broilers per experimental group. Within the same row, means bearing distinct superscript letters (a, b, c) indicate statistically significant differences (*p* < 0.05).

## Data Availability

The 16S rRNA sequencing data have been deposited in the NCBI Sequence Read Archive under BioProject accession number PRJNA1534161.

## Supplementary Materials

The following supporting information can be downloaded at: https://www.mdpi.com/article/10.3390/antiox15101317/s1. Table S1: The ingredient and nutrient level of the basal diet. Table S2: Composition of qRT-PCR system. Table S3: Program settings of qRT-PCR.
