# Supplementary material for: Dietary Sanguinarine Enhances Antioxidant Capacity and Intestinal Barrier Function in Yellow-Feathered Broilers via Modulation of Microbiota-Associated Butyrate Signaling
Source: Antioxidants (Basel). 2026 Oct 13;15(10):1317. doi: 10.3390/antiox15101317 (PMC13700857; doi:10.3390/antiox15101317)
Supplement: Supplementary file 1 [file antioxidants-15-01317-s001.zip › antioxidants-4565044-supplementary.pdf]

Table S1. The ingredient and nutrient level of the basal diet.

	Day 1–21	Day 22–38	Day 39–63
Feed ingredients (%)			
Corn	61.00	63.26	65.52
Soybean meal	32.00	28.00	24.00
Corn gluten meal	1.50	2.00	3.00
Soybean oil	1.40	2.50	3.50
Limestone	1.41	1.41	1.35
Dicalcium phosphate	1.33	1.33	1.33
DL-Methionine (99%)	0.18	0.15	0.12
L-Lysine-HCl (78%)	0.18	0.18	0.18
Wheat middling	0.11	0.17	0.00
Vitamin-mineral premix a	1.00	1.00	1.00
Calculated nutrient composition b			
ME (MJ/kg)	12.12	12.54	12.96
CP (%)	19.91	18.63	17.60
Lys (%)	1.09	1.00	0.92
Met (%)	0.51	0.46	0.42
Ca (%)	0.87	0.88	0.84
Available Phosphorus (%)	0.42	0.40	0.38

[a] The premix provided the following per kg of diet: VA, 6000 IU; VD3, 2000 IU; VE, 30 mg; VK3, 2 mg; VB1, 3 mg; VB2, 5 mg; pantothenic acid, 800 mg; choline chloride, 1500 mg; nicotinic acid, 30 mg; pyridoxine, 3 mg; folic acid, 500 mg; biotin, 0.2 mg; VB12, 1 mg; Fe, 100 mg; Cu, 8 mg; Mn, 100 mg; Zn, 100 mg; I, 0.42 mg; Se, 0.3 mg. Sodium Chloride, 3000 mg; b Values were calculated from data provided by Feed Database in China (2004).

Table S2 Composition of qRT-PCR system

**Reaction system composition**	**Dosage**
2 × ChamQ Universal SYBR Qpcr Master Mix	10 μL
PCR Forward Primer	0.4 μL
PCR Reverse Primer	0.4 μL
d H_2O (Sterilize distilled water)	7.2 μL
cDNA template 200 ng/μL	2 μL
Total	20 μL

Table S3 Program settings of qRT-PCR

Item	Program	cycle	temperature	time
Stage1	Pre-denaturation	1	95°C	30 seconds
Stage2	Circular reaction	40	95°C	10 seconds
			60°C	30 seconds
Stage3	Melting curve	2	95°C	15 seconds
			60°C	60 seconds
			95°C	15 seconds

2.1 Growth Performance

Body weight (BW) and feed consumption of each cage were recorded. Then, the average daily feed intake (ADFI), average daily weight gain (ADG), ratio of feed to weight gain (F: G) for the entire experimental period was calculated.

2.2 Analyses of serum biochemical indices

Immunoglobulin concentrations in serum (IgG, IgM, IgA) were determined with chicken-specific ELISA kits (Nanjing Jiancheng, Bioengineering Institute, Nanjing, China). The capacity for total antioxidant activity (T-AOC) and MDA content, along with the activities of total SOD (T-SOD), glutathione peroxidase (GSH-Px), catalase

(CAT), D-lactate acid (D-LA), diamine oxidase (DAO) and myeloperoxidase (MPO) were evaluated using assay kits (Nanjing Jiancheng, Bioengineering Institute, Nanjing, China) on a SpectraMax M5 (Molecular Devices, USA in accordance with the manufacturer's instructions).
